# Coronavirus Disease 2019 (COVID-19) Epidemic and Mental Health Status in the General Adult Population of Serbia: A Cross-Sectional Study

**DOI:** 10.3390/ijerph18041957

**Published:** 2021-02-17

**Authors:** Isidora Vujčić, Teodora Safiye, Bojana Milikić, Emina Popović, Draško Dubljanin, Eleonora Dubljanin, Jakša Dubljanin, Milanko Čabarkapa

**Affiliations:** 1 Institute of Epidemiology, Faculty of Medicine, University of Belgrade, Višegradska 26, 11000 Belgrade, Serbia; 2 Faculty of Medical Sciences, University of Kragujevac, Svetozara Markovića 69, 34000 Kragujevac, Serbia; teodoras0306@gmail.com (T.S.); bmilikic84@gmail.com (B.M.); chupa93@hotmail.com (E.P.); drjale81@gmail.com (J.D.); 3 Department of Children’s Healthcare, Community Health Center Petrovac na Mlavi, Moravska 2, 12300 Petrovac na Mlavi, Serbia; 4 Department of Pulmonology, University Clinical Hospital Center Zvezdara, Dimitrija Tucovića 161, 11120 Belgrade, Serbia; draskodubljanin@gmail.com; 5 Institute of Microbiology and Immunology, Faculty of Medicine, University of Belgrade, Dr Subotića 1, 11000 Belgrade, Serbia; eleonoraratkov@yahoo.com; 6 Department of Cardiology, University Clinical Hospital Center Zemun, Vukova 9, 11080 Belgrade, Serbia; 7 Department of Psychology, Faculty of Philosophy, University of Belgrade, Čika Ljubina Street 18–20, 11000 Belgrade, Serbia; cabarkapa.milanko@gmail.com

**Keywords:** COVID-19, coronavirus, DASS-21, depression, anxiety, stress, mental health, Serbia, psychological impact, epidemic

## Abstract

Since its outbreak, coronavirus disease 2019 (COVID-19) has rapidly spread throughout the world. The Serbian government declared a state of emergency on 15 March 2020, implementing some of Europe’s strictest measures to combat the pandemic. The aim of this study was to determine the impact of the COVID-19 epidemic on the mental health of the general adult Serbian population and to identify associated factors during the state of emergency and lockdown. Data were collected with a snowball sampling method between 23 March and 25 April 2020, by using an online questionnaire. Multiple ordinal regression was performed to establish the associations between socio-demographic characteristics, self-estimated health status, and depression, anxiety, and stress. Out of 1057 participants included in the study, 28.9%, 36.9%, and 38.1% reported moderate to severe depression, anxiety, and stress symptoms. Uneasiness related to COVID-19 news, the feeling of helplessness, likeliness of impending death, and presence of COVID-19 symptoms were associated with higher depression, anxiety, and stress scores. Current smoking status was associated with a higher risk of depression and stress. Students had a significantly higher level of depression and stress, while older age was protective against anxiety and stress. Higher socioeconomic status was significantly associated with lower levels of depression, anxiety, and stress.

## 1. Introduction

Originating as a cluster of unexplained cases of pneumonia in Wuhan, in December 2019, the outbreak of the novel coronavirus disease 2019 (COVID-19) has reached the level of a pandemic and caused a major public health crisis worldwide [1,2]. In order to slow the spread of disease and prevent health systems from becoming overwhelmed, many countries have implemented restrictions on population movement and full or partial lockdowns [3]. The government of Serbia declared a state of emergency on 15 March 2020, nine days after the first COVID-19 case was officially registered, implementing some of Europe’s strictest measures to combat the pandemic such as 12 h and weekend police-enforced curfew, strict bans on movement (especially people over 65), and shutting borders. Kindergartens, schools, universities, and cultural institutions were completely suspended, and students were learning via TV, staying indoors for days, weeks, or months [4]. Training and sports activities were suspended, too. On 12 March, Government’s Crisis Staff began to hold daily press conferences devoted to the coronavirus crisis. People were sitting home and watching TV, and social media played a major role in sharing news and connecting people. On 31 March, 2020, “Telekom”, the largest mobile service provider in the country, sent the following text message to all users: ‟The situation is dramatic. We are approaching the scenarios seen in Italy and Spain. Please, stay home” [5]. Although government-implemented measures were essential for containing the spread of the COVID-19, the disruption of a normal life during the state of emergency represents a serious threat to the mental health and well-being of the general population, as was shown in previous studies [6,7,8]. Panic and fear of disease, together with lockdown and physical distancing might lead to social isolation, loss of income, loneliness, inactivity, limited access to basic services, increased exposure to food, alcohol, and online gambling, and decreased family and social support, especially in older and vulnerable people [9]. A study conducted in China showed a high prevalence of mental health problems during the COVID-19 outbreak, which was positively associated with frequent social media exposure [10]. Several studies were conducted among the Serbian population previously investigating different aspects of COVID-19 on mental health, including studies investigating the levels of stress and resilience among academic medical staff [11], the mental health of medical personnel [12], and public trust and media influence on anxiety and depression levels among skilled workers [13], and there was a comparative study between Serbia, Lebanon, Italy, and Portugal of public reactions to the outbreak [14]. The first three mentioned Serbian studies were focused on selected population groups. The main goal of the fourth mentioned comparative study was to analyze anxiety levels related to COVID-19 in four countries that differ in culture, population size, and the age structure [14]. In contrast, our study has examined the psychological impact of the COVID-19 epidemic and lockdown on the general population of Serbia.

Taking all of this into account, the aim of this study was to determine depression, anxiety, and stress experienced by the general adult Serbian population and to identify associated factors during the state of emergency and lockdown.

## 2. Materials and Methods

### 2.1. Study Design and Participants

We adopted a cross-sectional study design. The study was conducted in accordance with the Declaration of Helsinki from 1975 (revised in 2013) [15], and the Institutional Review Board of the University of Belgrade, Faculty of Philosophy, Department of Psychology (Approval number: #2020-30) approved the study.

The required sample size was calculated to 909 for a five-category proportional odds model based on cumulative odds ratios of 1.30, 1.55, 1.90, and 2.52 estimated from previous studies, test power of 0.8, and two-sided α level of 0.05 [16,17,18]. To minimize social interactions, as recommended by the government of Serbia, an online anonymous questionnaire in the Serbian language with informed consent, demographic data, COVID-19-related questions, and the Depression, Anxiety and Stress Scale—21 items (DASS-21) was created using the Google Docs platform. We used a snowball sampling method by sharing the questionnaire link on Facebook and Viber medical community groups (internal medicine residents, health workers, psychologists, psychotherapists, and PhD students of medical sciences) that we are a part of, and encouraged others to share the link on various social networking platforms. Data collection took place from 23 March, 2020 (8 days after declaring the state of emergency in Serbia) until 25 April 2020 (still in a state of emergency).

To be eligible for the survey, respondents must have been aged 18 years or older and residents of the Republic of Serbia. Duplicate records were identified through examining the answers and were excluded. The respondents who did not complete the online questionnaire were also excluded from the analysis.

### 2.2. Measures

The online questionnaire included a brief study description and invitation, demographic data such as age, sex, employment status, profession and current occupation, country of residence, city of residence, the highest level of education, marital status, and religious beliefs, and socioeconomic status. Age was categorized into three groups: young adults (age 18–35), middle-aged adults (age 36–55), and older adults (age 56–86). Profession/occupation for each participant was categorized as a white-collar profession if it consisted mainly of office-related work or blue-collar profession if it consisted mainly of manual labor. Cities, where participants lived, were ranked according to population based on Serbian national registry data from 2011 [19] into Belgrade as a capital city, “largest cities” (Novi Sad, Nis, Kragujevac, Leskovac, Subotica, Krusevac, Kraljevo, and Pancevo), while the rest were grouped together and classified as “smaller cities”.

The second part of our questionnaire included questions related to general health issues and COVID-19 psychological impact, such as self-estimated general health status, presence/absence of chronic diseases, type of chronic disease present, current smoking status, self-estimated level of uneasiness related to COVID-19 news, fear of contracting COVID-19, feeling of helplessness, the likeliness of impending death, presence of COVID-19 symptoms, and awareness of being infected, or not, with SARS-CoV-2. Some health issues and fear of disease were measured on a 5-point response scale where category 5 represented “strongly agree” and category 1 represented “strongly disagree”. To facilitate data analysis, we collapsed responses “somewhat agree” and “agree completely” into “agree” and “somewhat disagree” and “disagree completely” into “disagree”.

The third part of our questionnaire was used to assess the mental health status of participants and included the standardized DASS-21 questionnaire. The DASS-21 questionnaire consists of 21 statements divided into three subscales, one for depression, one for anxiety, and one for stress, with each containing 7 statements. Participants selected how well they agreed with each statement for the past 7 days. Answers for each statement are categorized on an ordinal scale and have the following values: 0 for not at all, 1 for somewhat, 2 for quite often, and 3 for always. The sum of 7 scores of each subscale multiplied with 2 determines its final score. This questionnaire was shown to accurately measure levels of depression, anxiety, and stress during the COVID-19 outbreak in other countries, as well as during previous pandemics [20,21,22]. This questionnaire was also previously validated on the Serbian student population with reported Cronbach’s alpha values of 0.92 for the entire DASS-21 scale, 0.85 for the depression subscale, 0.81 for the anxiety subscale, and 0.84 for the stress subscale [23].

The depression score was calculated by adding scores from questions 3, 5, 10, 13, 16, 17, and 21 and multiplying it with 2. The anxiety score was calculated by adding scores from questions 2, 4, 7, 9, 15, 19, and 20 and multiplying it with 2. The stress score was calculated by adding scores from questions 1, 6, 8, 11, 12, 14, and 18 and multiplying it with 2. The final depression score was divided into 5 categories: normal (score 0–9), mild depression (score 10–13), moderate depression (score 14–20), severe depression (score 21–27), and extremely severe depression (score 28+). The final anxiety score was similarly divided into 5 categories: normal (score 0–6), mild anxiety (score 7–9), moderate anxiety (score 10–14), severe anxiety (score 15–19), and extremely severe anxiety (score 20–42). The final stress score was also divided into 5 categories: normal (score 0–10), mild stress (score 11–18), moderate stress (score 19–26), severe stress (score 27–34), and extremely severe stress (score 35–42). We have adopted ranges of each subset as recommended by the original authors (Lovibond and Lovibond) of the DASS-21 scale [24]. The same categorization was also used in other published studies [22,25,26].

### 2.3. Statistical Analysis

Statistical analysis was performed using SPSS Statistics Software (IBM SPSS Statistics for Windows, Version 22.0, Armonk, NY, USA). Numerical variables (age numerical, depression score, anxiety score, and stress score) were reported as means ± standard deviations, while frequencies and percentages were calculated for categorical variables. We used multiple ordinal regression to determine log-odds (Wald test estimates) and odds ratios (ORs) with confidence intervals (CIs) of 18 independent variables for each of the three DASS-21 subscales. Since independent variables in all three models were categorical, they were analyzed as factor variables. The lowest category of each ordinal scale was used as a reference variable (ref. var.). All tests were two-tailed, and the statistically significant probability level was *p* < 0.05.

## 3. Results

A total number of 1184 respondents completed the questionnaire, surpassing our calculated sample of 909. From this, 73 respondents who were not residents of the Republic of Serbia and 54 respondents younger than 18 years were excluded from the analysis, leaving a final sample of 1057 (89.3%) respondents from 146 different cities and towns in Serbia for analysis.

### 3.1. Demographic Characteristics of the Analyzed Sample

The mean age of respondents was 36.06 ± 14.95 (age range of 18–88 years), and the majority were females (67.7%). Less than one-third of participants were from Belgrade (28.7%), and the majority (46.5%) were from smaller cities. Out of analyzed respondents, the majority were employed (48.2%), white-collar professions (66.1%), with high school education (43.6%), single (51.9%), with a high level of religious belief (46.2%), and with average socioeconomic status (51.6%). The demographic characteristics of our sample are listed in Table 1.

### 3.2. Self-Estimated General Health Status and COVID-19 Psychological Impact

When it comes to self-estimated general health status, the majority of respondents did not report any chronic disease (74.3%), rated their general health status as good (46.5%), and were nonsmokers (63.2%). The psychological impact of the COVID-19 questionnaire showed that 527 respondents (49.8%) felt uneasiness related to COVID-19 news, 338 respondents (31.9%) had a fear of contracting COVID-19 disease, 330 respondents (31.3%) had a feeling of helplessness, and 85 respondents (8.1%) felt likeliness of impending death. The majority of respondents stated that they did not have COVID-19 symptoms (85.1%) and that they were not currently infected with SARS-CoV-2 (63.5%), while the rest did not know (36.5%) whether they were infected, or not. Nobody stated that they were infected with SARS-CoV-2. The self-estimated general health status and COVID-19 psychological impact of our sample are shown in Table 2.

### 3.3. DASS-21 Final Scores

The depression subscale of DASS-21 showed that 613 respondents (58.0%) had normal scores, while 144 (13.6%) had severe and extremely severe depression scores. The anxiety subscale of DASS-21 showed that 587 respondents (55.5%) had normal scores, while 212 (20.1%) had severe and extremely severe anxiety scores. The stress subscale of DASS-21 showed that 370 (35.0.5%) respondents had normal scores, while 199 (18.8%) had severe and extremely severe stress scores (Table 3).

### 3.4. Ordinal Regression Models

Ordinal logistic regression of all three DASS-21 subscales showed statistically significant improvement in fit relative to the null, or intercept only, model (depression: χ^2^ = 308.093, *p* = 0.000; anxiety χ^2^ = 423.590, *p* = 0.000; stress χ^2^ = 449.951, *p* = 0.000). Goodness-of-fit test showed that all three models are a good fit to the data (depression: Pearson *p* = 0.725; deviance *p* = 1.000; anxiety: Pearson *p* = 0.803; deviance *p* = 1.000; stress: Pearson *p* = 1.000; deviance *p* = 1.000; in all cases, *p* > 0.05). The depression model explains between 25.3% and 27.6% variance of dependent variable, the anxiety model explains between 33.0% and 35.8% variance of dependent variable, and the stress model explains between 34.7% and 36.6% variance of dependent variable (Cox and Snell pseudo R-squared: 0.253, 0.330, and 0.347, respectively; Nagelkerke pseudo R-squared: 0.276, 0.358, and 0.366, respectively). Based on the test of parallel lines, assumption of proportional odds was satisfied in all three models (depression: *p* = 0.937; anxiety: *p* = 1.000; stress: *p* = 0.074).

#### 3.4.1. Depression

Student status (OR 1.675; *p* = 0.045), current smoking (OR 1.337; *p* = 0.033), uneasiness related to COVID-19 news (OR 1.606; *p* = 0.024), feeling of helplessness (OR 3.139; *p* = 0.000), likeliness of impending death (OR 2.963; *p* = 0.000 for respondents who agreed and OR 1.808; *p* = 0.003 for respondents who were neutral), and presence of COVID-19 symptoms (OR 1.443; *p* = 0.046) were associated with higher levels of depression. Good general health (OR 0.316; *p* = 0.020) and higher socioeconomic status (average: OR 0.548; *p* = 0.004; above average: OR 0.449; *p* = 0.001) were associated with lower levels of depression (Table 4).

#### 3.4.2. Anxiety

Uneasiness related to COVID-19 news (OR 2.756; *p* = 0.000), fear of contracting COVID-19 (OR 2.987; *p* = 0.000 for respondents who agreed and OR 1.630; *p* = 0.006 for respondents who were neutral), feeling of helplessness (OR 1.971; *p* = 0.000), likeliness of impending death (OR 3.745; *p* = 0.000 for respondents who agreed and OR 1.722; *p* = 0.007 for respondents who were neutral), and presence of COVID-19 symptoms (OR 2.250; *p* = 0.000) were associated with higher levels of anxiety. People with above average religiosity had a higher level of anxiety on the borderline of statistical significance (OR 1.429; *p* = 0.050). Older age (OR 0.675; *p* = 0.046 for middle-aged and OR 0.443; *p* = 0.012 for older adults), good general health (OR 0.477; *p* = 0.019), and socioeconomic status above the average (OR 0.576; *p* = 0.023) were associated with significantly lower levels of anxiety (Table 5).

#### 3.4.3. Stress

The significantly higher levels of stress were associated with student status (OR 2.116; *p* = 0.001), average level of religious belief (OR 1.417; *p* = 0.038), presence of chronic disease (OR 1.410; *p* = 0.037), current smoking (OR 1.525; *p* = 0.001), uneasiness related to COVID-19 news (OR 2.685; *p* = 0.000 for respondents who agreed and OR 1.451; *p* = 0.043 for respondents who were neutral), fear of contracting COVID-19 (OR 2.178; *p* = 0.000), feeling of helplessness (OR 3.169; *p* = 0.000 for respondents who agreed and OR 1.515; *p* = 0.009 for respondents who were neutral), likeliness of impending death (OR 2.642; *p* = 0.000 for respondents who agreed and OR 1.639; *p* = 0.011 for respondents who were neutral), and presence of COVID-19 symptoms (OR 1.645; *p* = 0.004). Persons aged 56 or above (OR 0.385; *p* = 0.001) and those with socioeconomic status above average (OR 0.572; *p* = 0.011) had significantly lower levels of stress (Table 6).

## 4. Discussion

Our results showed that one month after the lockdown of the Serbian population and declaring the state of emergency due to the COVID-19 pandemic, 42.0% of study participants reported depression symptoms, 44.5% reported anxiety symptoms, and 65.0% reported stress symptoms.

The proportion of participants who had some form of depression in our study was higher than the estimated pooled prevalence of depression and depressive symptoms based on validated self-report questionnaires or structured interviews (27.0%) according to Wang J et al.’s systematic review and meta-analysis [27]. The prevalence of depression in our study was also higher than the estimated depression prevalence (33.4%) in a cross-sectional study of the Serbian and Italian student population that used the DASS-42 questionnaire and was conducted during a time when COVID-19 threat was not present [18]. A systematic review that investigated the effects of COVID-19 on psychological outcomes of the general population and included 19 studies from eight different countries (China, Spain, Italy, Irak, the US, Turkey, Nepal, and Denmark) pointed out that the prevalence of depressive symptoms ranged from 14.6% to 48.3% [28].

The prevalence of anxiety in our study was in accordance with that of similar studies that also used the DASS-21 questionnaire to assess the psychological impact of the COVID-19 epidemic in China (36.4%) [21], Northern Spain (39.8%) [29], and Spanish University of Valladolid workers and students (35.2%) [30]. However, it is interesting that in the Italian population, the prevalence of anxiety during the COVID-19 epidemic was low (18.7%), considering the epidemic impact this country sustained [22]. The prevalence of anxiety observed in our study was a little lower than the levels of anxiety reported in the Serbian and Italian student population during the time when COVID-19 was not present (45.6%) [18]. This can be explained by generally higher anxiety levels in the student population when compared with the general population, as was documented before [31].

Stress prevalence in our study was also higher than the reported prevalence in the China population (32.1%) [16], Basque Autonomous Community in Northern Spain (40.1%) [29], Spanish university community (40.3%) [30], and Italian general population (27.2%) [22]. The study conducted in Nepal revealed a very high prevalence of overall stress 81.9% using the Cohen Perceived Stress Scale [32]. Levels of depression, anxiety, and stress in our study were among the highest of all mentioned studies that assessed the psychological impact of the COVID-19 epidemic. The survey conducted between 27 April and 13 May 2020 among 678 participants mainly from the USA, Pakistan, and Canada showed that anxiety, stress, and depression were overwhelmingly prevalent in the world during the COVID 19 pandemic and the quarantine/social isolation [33]. High levels of stress, anxiety, and depression in our study were probably influenced by quarantine and lockdown measures, and several studies found that the duration of containment measures significantly influences the mental health and well-being of the general population [7,34], with significant differences in the prevalence of depression and anxiety between people in quarantine and people not in quarantine [35]. Media exposure could be one of the possible explanations for the high levels of stress in this study. Reasons for the higher prevalence of mental disorders in our population, when compared with other COVID-19-affected countries, could be explained by the additional stress that comes with lower income, potential instability of the region, general uncertainty, and relatively recent historical turmoil [36]. Therefore, it is necessary to recognize and examine people’s mental states in this unprecedented time [37].

Ordinal logistic regression analysis showed that factors significantly associated with higher depression scores in our study were student status, current smoking, uneasiness to COVID-19 news, the feeling of helplessness and likeliness of impending death, and the presence of COVID-19 symptoms. Several studies also pointed out that students experience higher levels of psychological distress [28,29,38] during the COVID-19 lockdown. The study conducted among the Australian adult population indicated that negative changes in smoking intake were associated with higher depression and also anxiety and stress symptoms since the onset of COVID-19 [39]. Smoking is associated with an increased risk of COVID-19 infection, and smokers are at higher risk of having adverse outcomes after becoming infected [40]. Additionally, significant positive associations between smoking and depression were observed [39]. Therefore, current smokers might perceive that they are at an elevated risk of physical hardship and recovery should they contract COVID-19 and thus feel more depressed. Several studies also showed that the presence of COVID-19 symptoms among study participants had an impact on depression level [21,41,42], which is also in accordance with results observed in our study. Factors associated with lower levels of depression in our study were good self-estimated health status and higher socioeconomic status. This is in accordance with other similar studies where poor health status and the presence of chronic illnesses, or other medical problems, were associated with higher levels of depression [21,22,42]. The study conducted in the USA indicated that lower income and having less than $5000 in savings were associated with a greater risk of depression symptoms during COVID-19 pandemic [43]. In our study, we found that socioeconomic status was inversely and significantly correlated with the risk of depression, with the highest risk for those with low socioeconomic status, while education level was not associated with depressive symptoms.

Factors associated with higher anxiety levels in our study were fear of contracting COVID-19, uneasiness to COVID-19 news, the feeling of helplessness, likeliness of impending death, and presence of COVID-19 symptoms. Factors associated with lower levels of anxiety were older age, good general health, and high socioeconomic status. Similar results were observed in the two studies on the Chinese population, where it was found that poor health, presence of chronic illnesses, and especially COVID-19 symptoms and breathing difficulties were predictors of higher anxiety levels [21,41]. It has been shown that fear of death not only predicts anxiety related to COVID-19 but also play a causal role in various mental health conditions [44]. Research has shown that people who are in isolation and quarantine experience significant levels of anxiety, anger, confusion, and stress, and that frequent media exposure may cause distress [37]. The study conducted in Serbia among skilled workers during the COVID-19 outbreak revealed that the majority of respondents were disturbed by the information distributed on public media and more than half of participants expressed their opinion that there was a lack of available information in public and felt disturbance accordingly [13]. Several studies, including ours, have reported lower rates of anxiety in older age groups compared with younger ones, probably because younger people spend more time on social media and other news outlets and constant exposure to COVID-19 news has been associated with increased levels of distress [45]. The study conducted in Germany indicated that frequency, duration, and diversity of media exposure were positively associated with more depressive and anxiety symptoms and participants with pre-existing fears seem to be particularly vulnerable to mental distress [46].

Several factors had a significant impact on higher levels of stress in our study, such as student status, average religiosity, current smoking, presence of chronic disease, the fear of contracting COVID-19, uneasiness to COVID-19 news, feeling of helplessness, the likeliness of impending death, and the presence of COVID-19 symptoms. Factors associated with lower levels of stress were older age and socioeconomic status above average. Student populations were repetitively reported to be at risk of higher stress level due to school closures, cancelation of social events, lower study efficiency with remote online courses, and postponements of exams [28,47]. The study conducted in the UK indicated that a quarter of current smokers have increased their smoking during this public health crisis and that mental health status and psychosocial well-being were strongly associated with tobacco consumption [40]. The two Chinese studies [21,41] had shown an association between the presence of COVID-19 symptoms and self-reported poor health with higher levels of stress. The study conducted among the general Lebanese population found that the presence of both COVID-19 fear and financial hardship significantly increased stress and anxiety [48], which is in line with our study results. In our study, average level of religious belief had a significant impact on stress symptoms, and a high level of religious belief had a significant impact on anxiety symptoms. A possible explanation for this finding could be that coronavirus-related anxiety was associated with an increased strengthening of religious beliefs, as was shown in the study by Rigoli [49].

Although levels of depression, anxiety, and stress were prevalent in our population during the state of the emergency, the study of Ocal et al. showed that Serbian respondents had higher resilience to COVID-19 than those from other countries (Lebanon, Portugal, and Italy), shown in the total emotional reactions scores as well as in the anxiety, stress, and depression dimensions [14]. We already mentioned that the prevalence of anxiety observed in our study was a little lower than the levels of anxiety in our population before the COVID-19 outbreak. A possible explanation for these findings could be specific socio-cultural reasons, such as previous experience with the Yugoslav War period [14]. During the last three decades, our country has faced similar situations many times, but they were imposed only when necessary, for short periods of time, they were not obligatory and not related to an outbreak since the smallpox epidemic in 1972 [13]. Another explanation could be that our study sample overrepresented young people and females. However, we cannot lower the effect of emergency state and implemented measures on the psychological state of the Serbian population. All these measures influenced greatly the mental health of our population, as well as in other regions in the world [13]. Results from our study can be used to identify persons at greater risk of suffering from psychological distress and furthermore, to develop psychological interventions and to implement adequate public mental health strategies. All smokers should also be encouraged to quit smoking during this critical time, providing them behavioral support. Health promotion strategies should be used to adopt or maintain positive health-related behaviors in the population.

The strength of this study could be the timing of data collection relative to lockdown restrictions in Serbia. There are several limitations of this study that must be considered when evaluating results. The major limitation is cross-sectional study design as it cannot determine causal relationships between studied variables. To overcome this problem and to better understand casual relationships, a longitudinal study is needed in order to validate our results. Because we used a self-reporting questionnaire in data collection, self-reporting bias may be present [39]. Another limitation that must be considered is our sampling method. The snowball sampling method is not random and has certain tendencies towards various biases [40]. For example, people without a computer, internet access, or social media accounts could not be recruited into the sample. Additionally, people with few social media friends and connections were less likely to be recruited. Although our sample size is relatively large (the study recruited more respondents than required by calculated sample size) and study participants have a wider geographical distribution, the sample may not be representative either by age or by gender and so our findings may not be generalized to the entire population. Probably, the sample overrepresented young people and females as we already mentioned. However, older adults might be more susceptible to the disease and thus feel more anxious about the disease.

More studies are needed to better assess mental disorders within specific populations, to discover causal relationships and to define measures needed to prevent, as well as to treat, mental disorders in the time of crisis.

## 5. Conclusions

Stress, anxiety, and depression were prevalent in the Serbian population during COVID 19 and the quarantine measures and lockdown.

Uneasiness to COVID-19 news, the feeling of helplessness, likeliness of impending death, and presence of COVID-19 symptoms were significantly associated with depression, anxiety, and stress levels in our study. Current smoking status was associated with a higher risk of depression and stress. Fear of disease and religious beliefs were associated with higher anxiety and stress scores. Students had a higher level of depression and stress, while older age was protective against anxiety and stress. The presence of chronic disease was associated with higher stress levels, while good health was associated with lower levels of depression. Higher socioeconomic status was significantly associated with lower levels of depression, anxiety, and stress.

## Figures and Tables

**Table 1:** Socio-demographic characteristics of study participants.

Variable	Category	Total Number (*N*)	Percentage (%)
Age Categorical	Young Adults (18–35)	603	57.0
	Middle-Aged Adults (36–55)	303	28.7
	Older Adults (56–86)	151	14.3
Sex	Males	341	32.3
	Females	716	67.7
Working status	Student	304	28.8
	Employed	509	48.2
	Unemployed	154	14.6
	Retired	90	8.5
Type of profession	White-Collar	699	66.1
	Blue-Collar	358	33.9
City of residence	Belgrade	303	28.7
	Bigger cities *	263	24.8
	Smaller cities	491	46.5
Education Level	Elementary school	49	4.6
	High school	461	43.6
	Bachelor	88	8.3
	University/Master/PhD	459	43.4
Marital status	Single	549	51.9
	Married	392	37.1
	Divorced	80	7.6
	Widowed	36	3.4
Religious belief	Low level	228	21.5
	Average level	341	32.3
	High level	488	46.2
Socioeconomic status	Below average	138	13.1
	Average	545	51.6
	Above-average	374	35.3

* Novi Sad, Nis, Kragujevac, Leskovac, Subotica, Krusevac, Kraljevo, Pancevo.

**Table 2:** Self-estimated general health status and coronavirus disease 2019 (COVID-19) psychological impact.

Variable	Category	Total Number (*N*)	Percentage (%)
Presence of chronic disease	Yes	272	25.7
	No	785	74.3
General health status	Very bad/Bad	61	5.8
	Average	209	19.8
	Good/Excellent	787	74..5
Current smoking status	Yes	389	36.8
	No	668	63.2
Uneasiness to COVID-19 news	Disagree	206	19.5
	Neither agree, nor disagree	324	30.7
	Agree	527	49.9
Fear of contracting COVID-19	Disagree	420	39.7
	Neither agree, nor disagree	299	28.3
	Agree	338	32.0
Feeling of helplessness	Disagree	465	44.0
	Neither agree, nor disagree	262	24.8
	Agree	330	31.2
Likeliness of impending death	Disagree	852	80.6
	Neither agree nor disagree	120	11.4
	Agree	85	8.0
Presence of COVID-19 symptoms	Yes	158	14.9
	No	899	85.1
Currently infected with severe acute respiratory syndrome coronavirus 2 (SARS-CoV-2)	Yes	0	0.0
	No	671	63.5
	I do not know	386	36.5

**Table 3:** Depression, Anxiety and Stress Scale—21 items (DASS-21) final scores.

Variable	Category	Total Number (*N*)	Percentage (%)
DASS-21 Depression Score Mean score: 9.94 ± 9.55	Normal	613	58.0
	Mild	138	13.1
	Moderate	162	15.3
	Severe	75	7.1
	Extremely severe	69	6.5
DASS-21 Anxiety Score Mean score: 8.44 ± 9.02	Normal	587	55.5
	Mild	80	7.6
	Moderate	178	16.8
	Severe	77	7.3
	Extremely severe	135	12.8
DASS-21 Stress Score Mean score: 16.48 ± 10.90	Normal	370	35.0
	Mild	284	26.9
	Moderate	204	19.3
	Severe	128	12.1
	Extremely severe	71	6.7

**Table 4:** Ordinal regression results for the depression model.

Characteristics		Depression		
	Log Odds	Odds Ratio	95% CI	*p*-Value
Age categorical, Young adults, ref. var.				
Middle-Aged Adults (36–55)	0.081	1.084	0.736–1.599	0.682
Older Adults (56–86)	0.078	1.081	0.606–1.928	0.791
Sex, females, ref. var.	0.235	1.265	0.944–1.696	0.116
Working status, Unemployed, ref. var.				
Student	0.516	1.675	1.011–2.777	0.045 *
Employed	−0.298	0.742	0.494–1.114	0.150
Retired	−0.562	0.570	0.278–1.168	0.125
Type of profession, Blue-collar, ref. var.	−0.005	0.995	0.698–1.419	0.978
City of residence, smaller cities, ref. var.				
Belgrade	0.099	1.104	0.808–1.509	0.533
Bigger cities	0.066	1.068	0.780–1.462	0.681
Education level, Elementary school, ref. var.				
High school	0.226	1.253	0.660–2.377	0.490
Bachelor	0.487	1.627	0.772–3.430	0.201
University/Master/PhD	0.471	1.602	0.788–3.254	0.193
Marital status, single, ref. var.				
Married	−0.194	0.824	0.563–1.206	0.319
Divorced	0.298	1.347	0.755–2.404	0.314
Widowed	0.554	1.741	0.799–3.794	0.163
Religious belief, Low level, ref. var.				
Average level	0.155	1.168	0.824–1.657	0.384
High level	−0.131	0.877	0.622–1.236	0.454
Socioeconomic status, Way below average, ref. var.				
Average	−0.602	0.548	0.365–0.820	0.004 *
Above average	−0.801	0.449	0.286–0.705	0.001 *
General health status, Bad, ref. var.				
Average	−0.226	0.798	0.455–1.397	0.429
Good	−0.714	0.316	0.268–0.894	0.020 *
Presence of chronic disease, No, ref. var.	0.076	1.078	0.759–1.533	0.674
Current smoking status, No, ref. var.	0.291	1.337	1.024–1.746	0.033 *
Uneasiness to COVID-19 news, Disagree, ref. var.				
Neither agree, nor disagree	−0.042	0.959	0.633–1.453	0.842
Agree	0.474	1.606	1.063–2.427	0.024 *
Fear of contracting COVID-19, Disagree, ref. var.				
Neither agree, nor disagree	−0.223	0.800	0.561–1.140	0.217
Agree	0.288	1.334	0.913–1.950	0.137
Feeling of helplessness, disagree, ref. var.				
Neither agree, nor disagree	0.306	1.358	0.952–1.936	0.092
Agree	1.144	3.139	2.206–4.466	0.000 *
Likeliness of impending death, Disagree, ref. var.				
Neither agree, nor disagree	0.592	1.808	1.225–2.669	0.003 *
Agree	1.086	2.963	1.810–4.850	0.000 *
Presence of COVID-19 symptoms, No, ref. var.	0.366	1.443	1.007–2.067	0.046 *
Currently infected with SARS-CoV-2, Do not know, ref. var.	0.043	1.044	0.793–1.374	0.758

* Statistical significance: *p* < 0.05; ref. var: reference variable

**Table 5:** Ordinal regression results for the anxiety model.

Characteristics	Anxiety			
	Log Odds	Odds Ratio	95% CI	*p*-Value
Age categorical, Young adults, ref. var.				
Middle-aged adults	−0.394	0.675	0.458–0.993	0.046 *
Older adults	−0.814	0.443	0.235–0.836	0.012 *
Sex, Females, ref. var.				
Males	−0.244	0.783	0.580–1.058	0.111
Working status, Unemployed, ref. var.				
Student	0.322	1.379	0.823–2.312	0.222
Employed	−0.088	0.915	0.604–1.388	0.678
Retired	0.101	1.106	0.513–2.387	0.797
Type of profession, Blue-collar, ref. var.	0.222	1.248	0.869–1.791	0.230
City of residence, Smaller cities, ref. var.				
Belgrade	−0.007	1.007	0.735–1.380	0.963
Bigger cities	0.259	1.295	0.943–1.779	0.110
Education level, Elementary school, ref. var.				
High school	−0.207	0.813	0.427–1.547	0.528
Bachelor	0.107	1.113	0.522–2.370	0.782
University/Master/PhD	−0.227	0.797	0.391–1.625	0.533
Marital status, Single, ref. var.				
Married	−0.025	0.976	0.664–1.433	0.900
Divorced	0.196	1.217	0.672–2.203	0.517
Widowed	0.148	1.160	0.488–2.755	0.737
Religious belief, Low level, ref. var.				
Average level	0.289	1.335	0.925–1.929	0.123
High level	0.357	1.429	1.000–2.041	0.050 *
Socioeconomic status, Below average, ref. var.				
Average	−0.122	0.885	0.575–1.364	0.580
Above average	−0.552	0.576	0.358–0.926	0.023 *
General health status, Bad, ref. var.				
Average	−0.164	0.848	0.470–1.531	0.585
Good	−0.740	0.477	0.257–0.885	0.019 *
Presence of chronic disease, No, ref. var.	0.221	1.248	0.881–1.767	0.212
Current smoking status, No, ref. var.	0.268	1.307	0.995–1.717	0.054
Uneasiness to COVID-19 news disagree, ref. var.				
Neither agree, nor disagree	0.423	1.526	0.978–2.380	0.063
Agree	1.014	2.756	1.773–4.286	0.000 *
Fear of contracting COVID-19, disagree, ref. var.				
Neither agree, nor disagree	0.489	1.630	1.149–2.312	0.006 *
Agree	1.094	2.987	2.051–4.350	0.000 *
Feeling of helplessness, disagree, ref. var.				
Neither agree, nor disagree	0.098	1.103	0.781–1.556	0.578
Agree	0.679	1.971	1.387–2.800	0.000 *
Likeliness of impending death, Disagree, ref. var.				
Neither agree, nor disagree	0.544	1.722	1.160–2.558	0.007 *
Agree	1.320	3.745	2.258–6.210	0.000 *
Presence of COVID-19 symptoms, No, ref. var.	0.811	2.250	1.573–3.217	0.000 *
Currently infected with SARS-CoV-2, Do not know, ref. var.	−0.128	0.880	0.669–1.157	0.360

* Statistical significance: *p* < 0.05.

**Table 6:** Ordinal regression results for the stress model.

Characteristics	Stress			
	Log Odds	*p*-Value	Odds Ratio	95% CI
Age categorical, Young adults, ref. var.				
Middle-aged adults	−0.298	0.743	0.526–1.049	0.091
Older adults	−0.954	0.385	0.220–0.673	0.001 *
Sex, Females, ref. var.	−0.189	0.828	0.634–1.081	0.165
Working status, Unemployed, ref. var.				
Student	0.750	2.116	1.335–3.353	0.001 *
Employed	−0.075	0.928	0.640–1.344	0.692
Retired	−0.136	0.873	0.439–1.734	0.697
Type of profession, Blue-collar, ref. var.	−0.021	0.979	0.710–1.350	0.897
City of residence, Smaller cities, ref. var.				
Belgrade	0.087	1.090	0.822–1.446	0.548
Bigger cities	0.150	1.161	0.872–1.548	0.307
Education level, Elementary school, ref. var.				
High school	−0.254	0.776	0.421–1.429	0.416
Bachelor	−0.352	0.704	0.347–1.426	0.329
University/Master/PhD	−0.188	0.829	0.423–1.622	0.583
Marital status, Single, ref. var.				
Married	0.116	1.123	0.795–1.585	0.510
Divorced	0.438	1.549	0.913–2.629	0.105
Widowed	0.329	1.390	0.659–2.931	0.388
Religious belief, Low level, ref. var.				
Average level	0.349	1.417	1.020–1.970	0.038 *
High level	0.219	1.245	0.903–1.715	0.181
Socioeconomic status, Below average, ref. var.				
Average	−0.197	0.821	0.556–1.212	0.321
Above average	−0.559	0.572	0.372–0.879	0.011 *
General health status, Bad, ref. var.				
Average	0.063	1.065	0.618–1.836	0.821
Good	−0.291	0.747	0.419–1.332	0.323
Presence of chronic disease, No, ref. var.	0.343	1.410	1.022–1.945	0.037 *
Current smoking status, No, ref. var.	0.422	1.525	1.194–1.947	0.001 *
Uneasiness to COVID-19 news, disagree, ref. var.				
Neither agree, nor disagree	0.372	1.451	1.011–2.081	0.043 *
Agree	0.988	2.685	1.852–3.892	0.000 *
Fear of contracting COVID-19, disagree, ref. var.				
Neither agree, nor disagree	0.102	1.107	0.816–1.503	0.516
Agree	0.779	2.178	1.538–3.085	0.000 *
Feeling of helplessness, Disagree, ref. var.				
Neither agree, nor disagree	0.415	1.515	1.111–2.065	0.009 *
Agree	1.153	3.169	2.287–4.391	0.000 *
Likeliness of impending death, disagree, ref. var.				
Neither agree, nor disagree	0.494	1.639	1.121–2.398	0.011 *
Agree	0.972	2.642	1.625–4.297	0.000 *
Presence of COVID-19 symptoms, No, ref. var.	0.498	1.645	1.175–2.302	0.004 *
Currently infected with SARS-CoV-2, Do not know, ref. var.	−0.075	0.928	0.724–1.190	0.556

* Statistical significance: *p* < 0.05.

## Data Availability

The data that support the findings of this study are available from the corresponding author (I.V.) upon reasonable request.
